# Self-efficacy beliefs in managing positive emotions: Associations with positive affect, negative affect, and life satisfaction across gender and ages

**DOI:** 10.3389/fnhum.2022.927648

**Published:** 2022-08-08

**Authors:** Mariagiovanna Caprara, Maria Gerbino, Minou Ella Mebane, Isabel M. Ramirez-Uclés

**Affiliations:** ^1^Department of Personality Psychology, Evaluation and Psychological Treatments, National University of Distance Education (UNED), Madrid, Spain; ^2^Department of Psychology, University of Rome “La Sapienza”, Rome, Italy; ^3^Faculty of Law, Università degli Studi Giustino Fortunato, Benvento, Italy

**Keywords:** self-efficacy, positive emotions, positive affect, negative affect, life satisfaction

## Abstract

Two studies were carried out on a Spanish population to explore the extent to which different self-efficacy beliefs in managing positive emotions are associated with common indicators of wellbeing, such as positive and negative affect or life satisfaction. The first study was conducted on 483 participants and attested to the factorial structure of three different self-efficacy beliefs: (a) perceived self-efficacy in expressing positive emotions; (b) perceived self-efficacy in retrieving memories of positive emotional experiences; and (c) perceived self-efficacy in using humor. The second study was carried out on 1,087 individuals between 19 and 80 years of age, and it provided evidence of the factorial invariance of the scales across age and gender. Furthermore, this latter study showed the association of self-efficacy in managing positive affect (SEMPA) with high chronic positive and low negative affect, and with high life satisfaction, controlling for gender and age. In younger participants, stronger associations were found between perceived self-efficacy in using humor and life satisfaction compared to older subjects. These findings may guide the design of interventions aimed at enhancing the potential benefits that could be drawn from the proper management of positive emotions.

## Introduction

A wide body of research has highlighted the crucial influence that individuals’ capacity to regulate their own emotions exerts on individual developmental pathways, on the quality of their interpersonal relationships and ultimately, on their successful social adjustment (Eisenberg and Spinrad, 2004; Saarni et al., 2006). Emotional regulation involves a wide variety of processes that are ultimately reflected in the expression, monitoring, and modification of both positive and negative emotions (Gross and Thompson, 2007). Much of the earlier research into emotional regulation focused on strategies that allow negative emotions like anger, sadness, and fear to be managed through cognitive reappraisal and expressive suppression (Aldao et al., 2010; Webb et al., 2012). However, later studies have pointed to the role that positive emotions like joy, love, and amusement may exert on cognitive performance, buffering the impact of negative experiences, and promoting wellbeing and optimal functioning (Lyubomirsky et al., 2005; Fredrickson, 2009; Guerra et al., 2012; Buonomo et al., 2019; Alexander et al., 2021; Catalino and Tov, 2022). In particular, research has emphasized the benefits of positive affect on psychological wellbeing and health, and the importance of regulatory skills that enable individuals to properly manage their positive emotional experiences (Lyubomirsky et al., 2005; Fredrickson, 2009). It is well known that the effectiveness of such emotional regulatory skills relies also on the beliefs that people hold about their capacity to master the challenges and the demands associated with various emotions, and the benefits obtained (Caprara, 2002; Bandura et al., 2003). Indeed, the regulation of emotions can be achieved, and it is effective, to the extent that people are convinced that emotions can be kept under their control (Tamir and Mauss, 2011; Ford and Gross, 2018).

### Self-efficacy beliefs and emotion regulation

Social cognitive theorists have argued that self-efficacy beliefs (i.e., individual’s beliefs about his/her own capacity to reach one’s own goals by orchestrating specific courses of action) play a special role in the regulation of emotions, affecting thought and actions in such a way that enables people to select and implement effective regulatory strategies (Bandura, 1997; Caprara, 2002). For example, the overall perceived capacity to control emotions in specific emotion-eliciting scenarios predicted college students’ positive and negative emotional experience over weeks and at 1 year (Tamir et al., 2007). By contrast, most of studies focused on the role of distinct self-efficacy beliefs in modulating overwhelming negative emotions, as well as on the appropriate experiencing and expression of positive emotions, especially in difficult situations (Bandura et al., 2003; Caprara et al., 2008). To this aim, the regulatory emotional self-efficacy scale (RESE) was developed, and it was used widely in several countries to assess those perceived capabilities (Bandura et al., 2003; Caprara et al., 2008; Gunzenhauser et al., 2013). This scale assesses individuals’ perceived self-efficacy in expressing positive emotions like joy, enthusiasm and pride, as well as, individuals’ perceived self-efficacy in managing anger/irritation and despondency/sadness.

There is a large body of evidence as to how people’s beliefs about their capacity to mitigate negative affect and to express positive emotions influence different facets of successful development and of social adaptation (Bandura et al., 2003; Caprara et al., 2010a; Lightsey et al., 2013; Milioni et al., 2015; Gerbino et al., 2018). For example, findings have shown that people’s confidence in their capacity to manage negative emotions can counteract depression and delinquency, while fostering life satisfaction (Bandura et al., 2003; Caprara et al., 2020). Likewise, people’s confidence in their capacity to express positive emotions is positively associated with self-esteem, optimism, prosociality, emotional stability, happiness, and contentment in daily-life situations (Caprara and Steca, 2005, 2006; Caprara et al., 2013b; Bassi et al., 2018). Several studies have documented how higher levels of perceived regulatory emotional self-efficacy are associated with indicators of wellbeing at different ages (e.g., Busseri, 2018).

### Perceived self-efficacy beliefs in managing positive emotions

A variety of studies have shown that the activation and the expression of positive emotions plays a crucial role in fostering wellbeing and successful adjustment, either directly or in conjunction with individual’s management of negative emotions, through self-efficacy beliefs. As such, positive emotions can be viewed as important sources of wellbeing and healthy adaptations, broadening the consequences of their management beyond their mere expression (e.g., Fredrickson, 2001; Fredrickson and Branigan, 2005; Dockray and Steptoe, 2010; Gloria and Steinhardt, 2016; Chang et al., 2019; Pressman et al., 2019). Individuals may take advantage of their positive emotions in different ways, so may be useful to examine the role of different self-efficacy beliefs related to different strategies of management of positive emotions. In particular, here we addressed three dimensions of self-efficacy in using positive emotions: (a) self-efficacy in expressing positive emotions; (b) self-efficacy in using humor; and (c) self-efficacy in using positive memories. Self-efficacy in expressing positive emotions has been defined as the perceived capability “to experience and to allow oneself to express positive emotions such as joy, enthusiasm, and pride in response to success or pleasant events” (Caprara et al., 2008). This dimension has been examined in different studies, many of them conducted on young adults. Overall, people who feel more capable of expressing positive emotions appear to be less inclined to use maladapted strategies of emotional regulation, such as suppression (Gunzenhauser et al., 2013), they experience more wellbeing due to their higher level of perceived social capacities at different ages (Caprara and Steca, 2005, 2006), and they are more prone to help and care for others because of their high levels of perceived empathic self-efficacy (Alessandri et al., 2009). Indeed, young adults who perceived themselves to be more capable of expressing their positive emotions on average reported a higher degree of happiness and contentment over time (Bassi et al., 2018). Longitudinal findings across early adulthood found that when self-efficacy beliefs in expressing positive emotions are enhanced from late adolescence to early adulthood, there was a less intense loss of self-esteem during this transitional period (Caprara et al., 2013a). Finally, recent findings, addressing the pandemic, corroborated that strong self-efficacy beliefs in the expression of positive emotions were associated with less depression and anxiety, also during a critical phase (Thartori et al., 2021).

However, effective management of positive emotions relies on the individual’s confidence in their capacity to not only express these but also, to savor, enhance, prolong, retrieve, and generate positive feelings (Bandura et al., 2003; Bryant et al., 2005; Caprara et al., 2008). Within this perspective, individuals’ capacity to retrieve positive emotional experiences, and to use humor when facing challenges and when coping with stressful situations has also been studied (Gerbino et al., 2018). Remembering positive autobiographical memories (e.g., Werner-Seidler et al., 2017) may be a powerful strategy to regulate emotions. The retrieval of memories of pleasant emotional experiences may in fact reactivate positive affect and counteract negative feelings, especially in situations that are difficult to handle (Bryant et al., 2005). For example, some experimental studies found that the activation of positive affect may counteract the negative emotions associated with a stress-response (Speer and Delgado, 2017), and that the activation of happiness and love by autobiographical memories reduces initial levels of induced anxiety (Demorest, 2020). As positive affect enhances an individuals’ resilience when coping with adversity and facing new challenges, pleasant memories may reactivate positive emotions, offering a means to redress negative experiences and dampen negative feelings (Rusting and DeHart, 2000; Walker et al., 2003; Joormann and Siemer, 2004; Joormann et al., 2007; Holmes et al., 2008; Werner-Seidler and Moulds, 2012).

Extending this line of reasoning, social cognitive research has explored the beliefs individuals hold about their capacities to retrieve positive emotional experiences (Gerbino et al., 2018). Perceived self-efficacy in taking advantage of memories of positive experiences (SE/MEM) consists in believing that one possesses the necessary skills to use past joyful emotional experiences to face stressful events (e.g., being able to recall positive memories when facing difficulties, being able to remember great experiences from the past when you are unhappy, remembering prior accomplishments when confronting new challenges: Gerbino et al., 2018). Findings have attested the unique association of these beliefs with indicators of wellbeing (strong positive affect and life satisfaction) and with low levels of negative affect in young adults from different western countries.

Humor can serve as an effective strategy to attenuate negative feelings and to help cope with challenges or stressful situations (Samson and Gross, 2012). Humor may moderate negative emotions by distancing unpleasant experiences and reducing the attentional resources assigned to negative events or fearful anticipations. Furthermore, humor may help people reappraise and reframe potentially stressful events in less threatening and harmful ways, thereby altering their emotional impact (Martin et al., 1993; Lefcourt et al., 1995; Strick et al., 2009). Humor may also foster positive interpersonal relationships (Martín, 2001; Kuiper, 2012), with humorous people attracting friends more easily and therefore, potentially benefiting from having a network of supportive relationships that promote and reflect their wellbeing (Fredrickson and Losada, 2005). To further explore the perceived capacities related to the emotional regulation of positive affect, the self-efficacy beliefs associated with using humor as a medium to overcome difficulties were assessed (Gerbino et al., 2018). Perceived self-efficacy belief in making positive use of one’s own sense of humor (SE/HUM) is the belief of being able to use humor to activate positive emotions (e.g., use humor to support your friends when they are sad and to deal with stressful situations). The association of perceived capabilities in using humor with high levels of positive affect was confirmed, as well as the association with a good quality of friendship in young adults from different countries (Gerbino et al., 2018).

### The neuroscience of self-efficacy, humor, and positive emotions

Although self-efficacy is one of the most extensively studied constructs in behavioral science and more than 40,000 documents on Scopus database use self-efficacy as a keyword, its neuroanatomical basis is poorly understood. Some studies have addressed the neurological basis of other constructs related to the “self,” such as self-esteem (Eisenberger et al., 2011; Agroskin et al., 2014), although self-efficacy beliefs differ widely from self-esteem (Bandura, 1997). Indeed, self-efficacy is a very specific construct, and it is reasonable to consider that different kinds of self-efficacy beliefs may be associated with different biological and neurological correlates. No studies to date have specifically addressed self-efficacy beliefs in relation to the regulation of positive emotions (i.e., in the use of humor, positive memories, and expressing positive emotions), although some studies have investigated the neural correlates of these strategies to regulate positive emotions.

With regards to humor, it is based on a complex set of biological processes that occur in the brain and nervous system (Martin, 2006). Humor can serve as a natural stress reliever and it can also improve the functioning of the cardiovascular, immunological and endocrine systems (Lefcourt et al., 1990; Bennett et al., 2003; Mobbs et al., 2003). Recently, neuroimaging techniques were employed to shed light on the affective, cognitive, and motor networks involved in humor processing. Event-related functional MRI (fMRI) studies showed that humor modulates activity in different cortical regions, engaging a network of sub-cortical regions that includes the nucleus accumbens, a key component of the mesolimbic dopaminergic reward system (Mobbs et al., 2003). Neuroimaging studies support the importance of the prefrontal and frontal lobes in integrating information during humor comprehension. Nevertheless, several studies showed strong activation of the left-hand side of the brain in response to verbal jokes. Indeed, widely distributed networks were seen to be involved in humor processing, including the medial temporal lobes, frontal lobes, language-related regions, ACC and others, such as the hippocampus, occipital cortex, temporal lobe, the limbic system and the amygdala, all regions and systems of venerable origin that are implicated in crucial survival function (Fry, 2002; Rozengurt, 2011).

With regards to the activation of positive memories, some studies have identified that positive memories dampen both the cortisol response in experimental stressful situation and morning cortisol level in adolescents exposed to early life stress (Speer and Delgado, 2017). In addition, positive memories have been associated with lower negative affect and self-cognition (Askelund et al., 2019). Furthermore, the experimental activation of positive memories was found to be associated with greater activity in brain regions previously related to the regulation of emotions, such as the bilateral ventrolateral prefrontal cortex (VLPFC) and corticostriatal regions associated with reward-processing (Speer and Delgado, 2017). Indeed, stronger VLPFC and dorsolateral prefrontal cortex connectivity was seen to be a function of enhanced feelings of positive emotions. Further studies found that activation of the ventromedial prefrontal cortex during stress was associated with more positive emotions during the recovery from stress (Yang et al., 2018).

In relation to the neural correlates of positive emotions, the use of neuroimaging techniques or electroencephalography (EEG) suggested that the formation and regulation of positive emotions, including happiness, is associated with a significant dampening of activity in the right prefrontal cortex and bilaterally in the temporoparietal cortex, as well as with enhanced activity in the left prefrontal regions. These phenomena were also associated with increased activity in the cingulate gyrus, inferior and ventral striatum, amygdala, and middle temporal gyri (Mak et al., 2009; Kringelbach and Berridge, 2010; Machado and Cantilino, 2017). In the context of neuropsychological theories, several studies have investigated how positive emotions can modulate cognitive control processing. Hence, electrophysiological techniques have shown that positive emotions can modulate cognitive control by increasing dopamine levels in frontal cortical areas (Xue et al., 2013), especially the ACC (Ashby et al., 1999; Dreisbach and Goschke, 2004).

### Aims of the studies

In the light of the importance of individuals’ perceived capacity to regulate positive emotions, we carried out two studies: the first one was set out to corroborate the validity of earlier measures of self-efficacy beliefs related to the expression of positive emotions, and that of more recent measures of self-efficacy beliefs about the retrieval of positive emotions and the use of humor (Gerbino et al., 2018). Subsequently, we performed a second study to assess the associations among those self-efficacy dimensions and wellbeing across genders and different ages. We did not expect gender or age to substantially modify the relationships between self-efficacy beliefs and wellbeing, in accordance with previous findings concerning humor, positive memories and wellbeing (Jiang et al., 2020). However, we cannot rule out that the perceived abilities in the three domains examined may have a slightly different relevance at different stages of life.

In the first study, the goal was to examine the factorial structure of the three different self-efficacy scales in a sample of middle-aged adults that assess: (a) the manifest expression of positive emotions (SE/POS); (b) the retrieval of positive memories to cope with current difficulties (SE/MEM); (c) and the use of humor to face critical situations (SE/HUM). Previous studies did not examine these three dimensions simultaneously (see Caprara et al., 2008; Gerbino et al., 2018), and we expected the findings to corroborate a model in which the three measures are traced to three co-related factors rather than to a model involving a unique first-order factor or three independent dimensions. It has been argued that expressing positive emotions like joy, enthusiasm and pride, as well as retrieving positive emotional experiences and making use of humor, are correlated, engaging skills derived from different experiences, producing different outcomes (Caprara et al., 2008), and representing distinct strategies for self-regulation and social adaptation.

The second study set out initially to corroborate the gender and age invariance of the three aforementioned measures of self-efficacy in managing positive emotions (comparing early, mid- and late adulthood), and to examine whether gender and age account for any significant difference in people’s self-efficacy beliefs. In this regard, women have been seen to have higher self-efficacy belief values than men in expressing positive emotions, although this difference tends to decline with age (Caprara et al., 2003). However, significant gender difference for self-efficacy beliefs has not been found in terms of using positive memories or using humor. Secondly, the aim was to corroborate and extend previous findings of how different self-efficacy beliefs that focus on managing positive emotions are associated with common indicators of wellbeing at different ages, such as life satisfaction, positive affect, and negative affect (Myers and Diener, 1995; Busseri, 2018), controlling gender, age and education in accordance with findings that indicate an association between education and wellbeing (Salinas-Jiménez et al., 2010).

## Materials and methods

### Participants and procedure

The first study was carried out on 483 middle-age adults aged between 30 and 45 years old (mean age = 37.923; SD = 4.08), 47.8% of whom were males. The participants were recruited from the psychology students at the Open University of Madrid (UDIMA) using a snowball sampling technique and they received course credits on enrollment. All the participants were born in Spain, and they were considered to be middle class and moderately-to-highly educated (4.6% were primary school graduates or lower, 10.2% were high school graduates, 24.9% had an undergraduate degree, and 60.4% had a graduate degree). The participants were informed that their responses would be treated anonymously, and that full confidentiality would be guaranteed throughout the research. The study was approved by the Institutional Review Board at the UDIMA.

In the second study, data were collected from a total of 1,087 adult participants (50.7% males) in the age range of 19–80 (*M* = 45.1, SD = 14.78), and they were divided into three age groups: young (20−40), middle-aged (41−60), and old (>61). The participants were recruited using the same procedure indicated above, they were all born in Spain, and they were middle class and moderately-to-highly educated (10.6% were primary school graduates or lower, 11.9% were high school graduates, 28.6% had an undergraduate degree, and 48.6% had a graduate degree). The study followed a similar procedure as the first study and the data came from a larger database that was in part used elsewhere (Caprara et al., 2020).

### Measures

In the first study, perceived self-efficacy in managing positive affect (SEMPA) was measured using the following three subscales: (a) Perceived self-efficacy in expressing positive emotions (SE/POS) was measured with four items of the RESE (Caprara and Gerbino, 2001; Caprara et al., 2008), addressing the individual’s perceived capacity to experience and allow themselves to express positive emotions like joy, enthusiasm, and contentment on a 5-point Likert scale, from 1 = not well at all to 5 = very well (e.g., “How well can you rejoice over your successes?”); (b) Perceived self-efficacy in taking advantage of memories of positive experiences (SE/MEM; Gerbino et al., 2018) was measured with four items addressing one’s own perceived capability to use positive memories when facing critical situations on a 5-point Likert scale from 1 = not at all capable to 5 = completely capable (e.g., “How well can you find comfort in remembering moments of joy when you find yourself in difficulty?”); (c) Perceived self-efficacy in the positive use of humor (SE/HUM; Gerbino et al., 2018) was measured with four items that assess the perceived self-efficacy in making positive use of one’s own sense of humor when in difficulty on a 5-point Likert scale from 1 = not at all capable to 5 = completely capable (e.g., “How well can you overcome embarrassing and difficult situations with playful jokes”). The Cronbach’s alpha value of the RESE was = 0.68, that of the SE/MEM was = 0.62 and the value for SE/HUM was = 0.75.

In the second study, the perceived SEMPA was again measured with the three scales: SE/POS, SE/MEM, and SE/HUM. As an indicator of wellbeing, life satisfaction was measured using five items of the Spanish version (Cabañero et al., 2004) of the Satisfaction with Life Scale (SWLS; Diener et al., 1985). The participants rated the extent to which they generally felt satisfied with life (e.g., “I am satisfied with my life”) on a 7-point scale, ranging from 1 (= *strongly disagree*) to 7 (= *strongly agree).* The Cronbach’s alpha of the SWLS was 0.83. Similarly, Positive and Negative Affect was assessed with the Spanish version (Ortuño-Sierra et al., 2015) of the positive and negative affect schedule (PANAS; Watson et al., 1988). This scale was developed to measure two higher-order dimensions of self-rated positive and negative affect, and it included ten items that measure positive affect (e.g., “active,” “attentive,” “enthusiastic,” and “excited”: α = 0.80) and ten items that measure negative affect (e.g., “afraid,” “hostile,” and “irritable”: α = 0.83).

In both studies, data regarding some socio-demographic variables were collected: age, gender, educational level (i.e., primary school or lower; high school; undergraduate; graduate degree, such as Master’s or Ph.D.).

## Results

### Study 1: Factorial structure of self-efficacy in managing positive emotions

With the aim of confirming the factorial structure of the three scales of self-efficacy in managing positive emotions, we conducted a preliminary exploratory factor analysis (EFA) on the 12 items of the three self-efficacy scales using Mplus 8.0 (Muthén and Muthén, 1998–2017). A three factor solution coherent with the three hypothesized dimensions (SE/POS, SE/MEM, and SE/HUM) produced the best fit (comparative fit index-CFI: one factor = 0.74; two factors = 0.86; three factors = 0.97), although one item of the SE/MEM scale (i.e., “How well can you remember your past successes when you are confronting new challenges”) cross-loaded onto two different factors with a low, yet similar loading coefficient (0.19 and 0.27). Given that a previous validation study already indicated that this item did not produce optimal loading in a Spanish sample (Gerbino et al., 2018), we decided to leave this out and thus, we conducted an EFA on the 11 remaining items, confirming the best fit of the three factors solution (CFI = 0.974).

**FIGURE 1:**
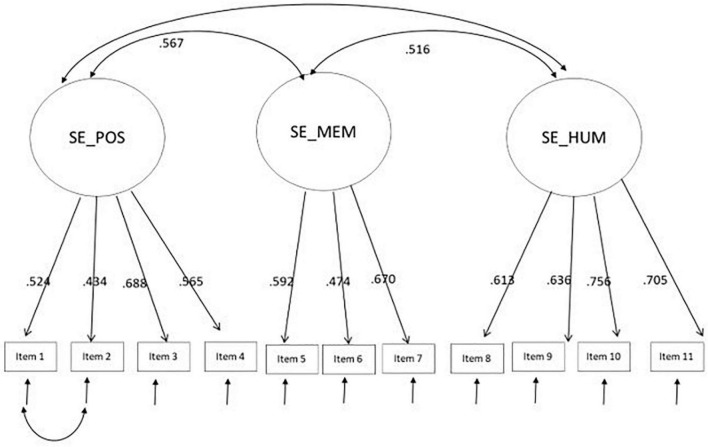
Path diagram of the factorial structure of self-efficacy in managing positive emotions.

We then further tested if the three factors structure of the scale fitted data well by performing a confirmatory factor analysis (CFA) on the 11 items. We tested and compared three alternative models (Table 1): (1) a one factor model hypothesizing that all items loaded on a single latent factor (Model 1); (2) a three factor oblique model in which the SE/POS, SE/MEM, and SE/HUM were considered as separate and correlated factors (Model 2); (3) a model with three orthogonal factors (Model 3). Of these, Model 2 with three oblique factors fitted the data in the sample well (see Figure 1 and Table 1) and produced a better fit than the other models. Before attaining these model fits, a sequential fit diagnostic evaluation analyses of each sample indicated one point of ill-fit due to error covariance in one pair of items, items 1 and 2. Thus, we added the covariance between these two items and the resulting fit of the model was satisfactory. The correlations between the three latent factors were moderate, ranging from 0.49 to 0.58.

**TABLE 1:** Fit indices for the confirmatory factor analysis (CFA) of the self-efficacy in managing positive affect (SEMPA) in the Spanish sample.

Models	χ^2^	*df*	*CFI*	*RMSEA*	*CI RMSEA*	*SRMR*	*AIC*
Model 1: Unique factor	337.54	44	0.73	0.12	[0.10–0.13]	0.078	14,176.24
Model 2: 1st order oblique factors	88.64	41	0.96	0.049	[0.035–0.063]	0.035	13,933.33
Model 3: 1st order orthogonal factors	193.99	43	0.86	0.085	[0.073–0.098]	0.12	14,034.69

For each sample, Model 1 refers to one factor with 11 items; Model 2 refers to two correlated, first-order factors (SE/MEM and SE/HUM, SE/POS each with four items); Model 3 refers to two orthogonal first-order factors (SE/MEM and SE/HUM, each with four items).

**FIGURE 2:**
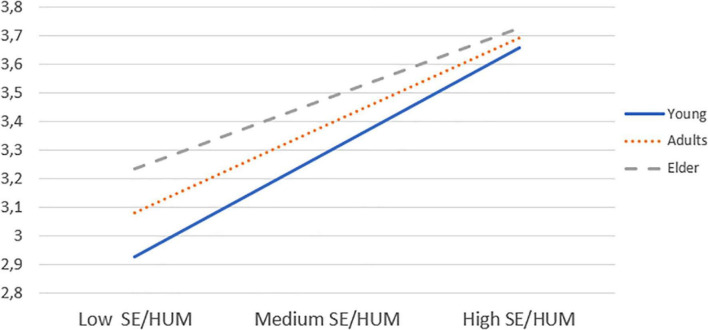
Plot of the interaction of self-efficacy beliefs in using humor and age in predicting life satisfaction.

### Study 2: Self-efficacy beliefs in managing positive emotions and their associations with indicators of wellbeing across gender and age

A multiple-group CFA was carried out to confirm the scale dimensionality by testing the measurement invariance across sex and ages (Mplus 8.0; Muthén and Muthén, 1998–2017). Measurement invariance was tested by running three consecutive and more restrictive nested models using the full information maximum likelihood (FIML) method of estimation (Vandenberg and Lance, 2000). Three models were tested: (1) configural invariance where the same pattern of fixed and free-factor loadings was imposed across the groups; (2) metric invariance where factor loading estimates were constrained equally across the groups; and (3) scalar invariance where both factor loadings and latent intercepts were constrained equally across the groups. A variety of goodness-of-fit indices were used to evaluate the model’s fit: (a) Chi-squared difference tests were used to compare the nested models, although the Chi-squared test is sensitive to sample size such that it is more likely to obtain a significant chi-squared value with a larger sample size (Kline, 1998); (b) CFI, employing a cut-off threshold < 0.95 (Hu and Bentler, 1999); (c) root mean square error of approximation (RMSEA), with a cut-off < 0.08; and (d) standardized root mean square residual (SRMR), also with a cut-off < 0.08 (Browne and Cudeck, 1993). Akaike information criterion (AIC) was used to compare non-nested models whereby the lower the AIC value the better the model’s fit (Tabachnick and Fidell, 2001). Furthermore, following previous recommendations (Chen, 2007) the following criteria were adopted: ΔCFI < 0.005 and ΔRMSEA < 0.01.

#### Gender invariance

The stability of the latent structures of SEMPA was investigated across gender and the fit indices for the gender invariance models were obtained (see Table 2). The model with factor loadings unconstrained to be equal across the sexes fit the data reasonably well, confirming configural invariance. Hence, the hypothesis of full metric invariance was then tested and accepted. Next, full scalar invariance was tested and although the change of the chi-squared value was significant, the ΔCFI was lower than 0.005 such that full scalar invariance was accepted.

**TABLE 2:** Fit indices for gender and age invariance of self-efficacy in managing positive emotions.

	χ^2^	*df*	*CFI*	*RMSEA*	*CI RMSEA*	*SRMR*	Δχ^2^	Δ*df*	*P*	Δ*CFI*
**Gender invariance**										
Model 1: Configural invariance	170.78	80	0.97	0.046	0.036–0.055	0.035				
Model 2: Metric invariance	180.64	88	0.97	0.044	0.035–0.053	0.040	9.86	8	0.28	0.001
Model 3: Scalar invariance	203.38	96	0.96	0.045	0.037–0.054	0.044	32.60	16	0.008	0.005
Model 3a: Partial scalar	195.89	95	0.97	0.044	0.035–0.053	0.043	15.26	15	0.033	0.003
**Age invariance**										
Model 1: Configural invariance	219.29	120	0.97	0.048	0.038–0.058	0.039				
Model 2: Metric invariance	242.18	135	0.96	0.15	0.037–0.056	0.049	22.89	15	0.086	0.002
Model 3: Scalar invariance	286.74	152	0.95	0.049	0.041–0.058	0.055	44.57	17	0.000	0.010
Model 3a: Partial scalar	277.51	151	0.96	0.048	0.039–0.057	0.055	35.33	16	0.004	0.007

In the age invariance, the intercept of the item 8 was released in the 35–59 group.

#### Age group invariance

We investigated whether the latent structure of SEMPA was replicated across the three age groups tested, defining the fit indices for the age invariance models (see Table 2). The model with factor loadings unconstrained to be equal across the age groups fit the data reasonably well, confirming configural invariance in all three groups. As such, the hypothesis of full metric invariance was tested and accepted. Next, full scalar invariance was tested but rejected. Following modification of the indices, we relaxed the equality constraint on the intercepts associated with item 9, measuring SE/HUM in the middle-age adult group. After this, the partial scalar invariance was tested and accepted.

#### Gender and age differences

After ascertaining the invariance of the scales, differences due to gender and age (early, middle, and older adulthood) in terms of the three self-efficacy beliefs were investigated. An overall MANOVA witnessed a significant effect of gender [*F*(1078, 3) = 7.865, *p* = 0.001, η^2^ = 0.021] but not of age [*F*(1078, 3) = 1.4131, *p* = 0.206; see Table 3]. The results of a univariate ANOVA showed that women reported a higher level of self-efficacy beliefs when expressing positive emotions than men, although the effect index (partial η^2^ = 0.011) indicated that the differences had little relevance. No significant interaction effect was found between age and gender [*F*(2154, 6) = 6.000, *p* = 0.10]. Regarding positive and negative affect, and life satisfaction, the overall MANOVA indicated a small significant effect of age [*F*(2158, 6) = 3.023, *p* = 0.0001, η^2^ = 0.008] but not of gender [*F*(1079, 6) = 0.979, *p* = 0.402]. In examining the univariate ANOVAs, a significant age difference was only evident for Life Satisfaction (η^2^ = 0.012) and a *post hoc* analysis conducted with the Tukey’s *B* test revealed that the older group aged 60–80 reported significantly greater life satisfaction than the two younger groups (See Figure 2).

**TABLE 3:** Gender and age differences in the dimensions of self-efficacy for the management of positive emotions and for indicators of wellbeing.

	All		Men		Women		Younger		Middle		Older		
	M	SD	M	SD	M	SD	M	SD	M	SD	M	SD	Gender and age effects
SE_POS	3.96	0.72	3.89	0.032	4.05	0.032	3.96	0.038	3.93	0.032	4.02	0.046	Gender: *F* = 12.16, *p* = 0.0001, η^2^ = 0.011 Age: *F* = 1.10, *p* = 0.33, η^2^ = 0.002
SE_MEM	3.36	0.79	3.36	0.036	3.36	0.035	3.33	0.042	3.35	0.036	3.40	0.051	Gender: *F* = 0.017, *p* = 0.896, η^2^ = 0.001 Age: *F* = 0.57, *p* = 0.57; η^2^ = 0.001
SE_HUM	3.50	0.79	3.54	0.036	3.46	0.035	3.55	0.042	3.50	0.035	3.45	0.051	Gender: *F* = 2.43, *p* = 0.12, η^2^ = 0.002 Age: *F* = 1.10, *p* = 0.33, η^2^ = 0.002
Life satisfaction	3.39	0.83	3.38	0.84	3.40	0.82	3.31	0.82	3.37	0.81	3.55	0.860	Gender: *F* = 0.001, *p* = 0.97, η^2^ = 0.0001 Age: *F* = 6.30, *p* = 0.001, η^2^ = 0.012, Older > Younger, Middle
Positive affect	2.95	0.51	2.93	0.51	2.97	0.52	2.91	0.55	2.95	0.49	2.95	0.570	Gender: *F* = 0.72, *p* = 0.40, η^2^ = 0.001 Age: *F* = 0.38, *p* = 0.97, η^2^ = 0.0001
Negative affect	1.97	0.56	1.96	0.55	1.99	0.56	2.01	0.55	1.97	0.55	1.94	0.570	Gender: *F* = 1.48, *p* = 0.23, η^2^ = 0.001 Age: *F* = 1.62, *p* = 0.20, η^2^ = 0.003

#### Associations among demographic variables, self-efficacy beliefs, life satisfaction, and positive and negative affect

After examining the age and gender differences in the three scales of self-efficacy beliefs in managing positive emotions, we examined the association among the above scales, socio-demographic variables and some indicators of wellbeing. Overall correlations showed that the correlations of demographic variables with self-efficacy beliefs and wellbeing indicators were weak (see Table 4), with old age associated with low education levels and high life satisfaction, being female associated with a high SE/POS, and a higher level of education associated with stronger positive affect. The correlations among self-efficacy beliefs related to the management of positive emotions were positive and moderate. Furthermore, self-efficacy beliefs showed significant, positive, and moderate correlations with both life satisfaction and positive affect, and significant but low and negative correlations with negative affect.

**TABLE 4:** Correlations among the socio-demographic variables, self-efficacy beliefs in managing positive emotions, and indicators of positive affect, negative affect, and life satisfaction.

	(1)	(2)	(3)	(4)	(5)	(6)	(7)	(8)	(9)
(1) Gender									
(2) Age	0.030								
(3) Education	–0.018	−0.26**							
(4) SE/POS	0.12**	0.011	0.054						
(5) SE/PMEM	–0.011	–0.018	0.048	0.36***					
(6) SE/HUM	–0.041	–0.055	0.058	0.35***	0.39***				
(7) Life satisfaction	0.014	0.095**	0.024	0.35***	0.35***	0.36***			
(8) Positive affect	0.031	–0.009	0.089**	0.44***	0.32***	0.40***	0.48***		
(9) Negative affect	0.026	–0.051	–0.042	−0.30***	−0.22***	−0.19***	−0.31***	−0.26***	

**p < 0.01; ***p < 0.001.

To examine the unique associations of the three different self-efficacy beliefs with life satisfaction, or positive and negative affect, a hierarchical regression was performed using the variables of age, gender, and education as predictors in step 1, the three self-efficacy beliefs in managing positive emotions in step 2, and the respective interactions of age and gender with the three self-efficacy beliefs in step 3. Education was considered as a control variable in the model. In line with previous studies (Cohen et al., 2003), lower-order and interactive terms were mean-centered to facilitate a correct interpretation of the lower-order terms and to decrease non-essential multicollinearity. With regards to control variables, our findings indicate that higher education was associated with a stronger positive affect (see Table 5). All three self-efficacy beliefs accounted for a significant portion of variance in life satisfaction and positive affect, while only SE/POS and SE/HUM accounted for part of the variance in negative affect. In addition, only the interactions of SE/HUM with gender and SE/HUM with age accounted for any significant variation in life satisfaction. To further examine the effect of the interaction terms, we ran a simple slope analysis, and we probed the effect of Self-Efficacy beliefs at low (–1 SD), medium and high (+1 SD) ages, as well as for males and females (Cohen et al., 2003). The results showed that SE/HUM was more positively related to life satisfaction, especially at a younger age (β = 0.47; *p* = 0.001) than in middle (β = 0.39; *p* = 0.001) and older adulthood (β = 0.31; *p* = 0.001). Finally, a simple slope analysis failed to confirm the significance of the interaction between SE/HUM and sex.

**TABLE 5:** Results of hierarchical regressions analyses (HRA) of socio-demographic variables and of self-efficacy in managing positive emotions on indicators of wellbeing.

	Life satisfaction			Positive affect			Negative affect	
	β	(SE)	*R*^2^ change	β	(SE)	*R*^2^ change	β	(SE)	*R*^2^ change
Step 1			0.012			0.009			
Gender	0.022	0.050		0.037	0.031				0.006
Age	0.006**	0.002		0.00	0.001		0.030	0.034	
Education	0.043	0.026		0.047**	0.016		−0.002	0.001	
							−0.032	0.017	
Step 2			0.21***			0.26***			
SE/POS	0.22***	0.036		0.21***	0.021				0.10***
SE/MEM	0.20**	0.032		0.081***	0.019		−0.19***	0.026	
SE/HUM	0.22***	0.033		0.15***	0.020		−0.075**	0.023	
Step 3			0.017***			0.06			
Gender × Age	−0.004	0.003		0.001	0.002				0.006
SE/POS × Gender	0.16	0.071		−0.015	0.043		0.001	0.002	
SE/MEM × Gender	0.072	0.063		0.023	0.038		−0.064	0.051	
SE/HUM × Gender	−0.18**	0.065		0.071	0.040		0.047	0.047	
SE/POS × Age	0.002	0.002		0.002	0.001		0.043	0.002	
SE/MEM × Age	−0.001	0.002		0.000	0.001		−0.023		
SE/HUM × Age	−0.007**	0.002		0.001	0.001		0.014		
Adjusted *R*^2^		0.24				0.27			0.11

**p < 0.01; *** *p* < 0.001. Sex was coded as 0 = men and 1 = women.

## Discussion

Overall, the findings of our studies confirm the role that self-efficacy beliefs may play in enabling individuals to regulate their emotions and to benefit from their emotional experiences. Furthermore, our results extend previous findings (Gerbino et al., 2018) regarding the internal structure and validity of the three scales designed to assess self-efficacy beliefs in relation to an individuals’ capacity to express positive emotions, retrieve memories of positive emotional experiences and use humor.

The first study corroborates previous findings, showing that the three scales can be traced to independent yet related latent dimensions. A CFA indicated that a three oblique factors model is the best to compare alternative models, using either a single first-order factor or three orthogonal factors. These results suggest that self-efficacy beliefs that are related to strategies that benefit from positive emotions might be traceable to distinct, although correlated latent dimensions. This is consistent with suggestions from recent research on self-efficacy beliefs related to the management of negative emotions like anger, sadness, fear, guilt, and shame (Caprara et al., 2020). Moreover, these data further strengthen the idea that emotional regulation is a complex domain, whereby different mental structures operate in concert, and distinct strategies may be required to adequately deal with the various manifestations of positive and negative affect. While special attention must be paid to understand what is common to all emotions, and what distinguishes positive and negative emotions, it is necessary to fully appreciate the uniqueness of each emotion in terms of its sources, manifestations, impact, and regulation. This also applies to self-efficacy beliefs, which can be traced to a common self-system that oversees the overall interactions of the individual with their environment, accounting for their sense of identity, continuity, consistency, and agency. However, in practice, learning, and reflecting upon experience alters an individual’s control over themselves and their environment (Bandura, 2008). Consequently, individuals hold different beliefs about their capacities and pursuits across the different domains of functioning and context.

Self-efficacy beliefs are generalized across functional domains, like motivation, cognition, and emotion. Yet even within the same active domain, individuals show different levels of confidence in their ability depending on the opportunities they have had to practice and demonstrate them (Bandura, 1997). Thus, it is common for people to be confident in their ability to deal with certain emotions, despite feeling uncertain with others. Hence, it should be no surprise that some individuals are more inclined to spread their joy and enthusiasm, while others more often use humor to amuse themselves and their fellows, and yet others remember good times and savor the good feelings that can be retrieved from them. As indicated for self-efficacy beliefs related to the management of negative emotions (Caprara et al., 2020), it is recommendable to pay attention to what is distinctive about self-efficacy beliefs relevant to the management of positive emotions. As such, interventions that enhance the benefits of positive emotions could be designed. Indeed, externalizing ones’ joy and enthusiasm, using humor and retrieving past memories are different ways to benefit from positive emotions that engage distinct mental processes, drawing on experiences that are accessible in a variety of manners. Moreover, while the retrieval of positive emotional experiences can be a source of intimate pleasure and satisfaction from within, the externalization of positive emotions and humor are primarily embedded in interpersonal relationships, and their effect on personal wellbeing might be mainly the result of their positive impact on others (Gerbino et al., 2018).

These findings do not allow us to establish whether those who are inclined to externalize their joy are also those who have a good sense of humor, and who are most inclined to rely on their positive emotional experiences to gain comfort and solace. Nevertheless, the moderate correlations of self-efficacy beliefs related to the management of positive emotions draw our attention to the distinctive effect of each of them, rather than what they have in common. Likewise, the correlations of the three indicators of wellbeing show that the mastery of beliefs associated with each of the three regulatory strategies can be effective, albeit distinctly. Feeling able to express ones’ own positive emotions, use humor, and reactivate pleasant memories all contribute to life satisfaction, allowing individuals to remain in a good mood, although they are less effective in mitigating negative emotions. Since this occurs across age and gender, further studies will be needed to clarify the reasons for the stronger impact of self-efficacy beliefs on expressing one’s own emotion as opposed to using humor and retrieving memories. Intuitively one may suspect that to ventilate ones’ own positive affect is more direct, spontaneous and easy than construing humorous situations or retrieving pleasant narratives.

The findings that older people report being more satisfied with their lives than younger people are consistent with earlier studies in which a positive association between aging and happiness has been shown, also known as “the paradox of wellbeing” (Carstensen et al., 1998; Mroczek and Kolarz, 1998; Kunzmann et al., 2000; Siedlecki et al., 2008; Stone et al., 2010). However, as aging is typically associated with worse physical health and more losses, one may wonder whether such declarations are due to a better calibration between aspirations and successful adaptation. Women indicate they are more confident than men in their capacity to express positive emotions, also corroborating previous findings. Indeed, it has been suggested that culture and socialization may participate in enabling females to express their positive affect better than males (Caprara et al., 2003, 2013b). Findings that confidence in one’s own capacity for humor is associated with life satisfaction, mainly in young people, may reflect the importance of being able to tell jokes and to say funny things at an age when it is crucial to make friends and to establish rewarding relationships (Martin et al., 2003).

In the physiological context of neuroscience, there is evidence implicating specific brain areas and neurotransmitters in humor and suggesting that certain brain areas may be responsible for controlling positive emotions. Nevertheless, there is still much ground to be covered if we are to understand the physiological elements that are responsible for modulating positive emotions, humor, and self-efficacy. Perhaps future efforts could first focus on observing the activity in the brain areas implicated in such modulation. In terms of the neuroscientific studies related to self-efficacy carried out to date, most consider the variables self-efficacy and self-esteem interchangeable, even though they are clearly different constructs. As such, we suggest that future research should use specific assessment instruments that allow the specific brain areas implicated in self-efficacy processes to be determined from a neuroanatomical, neurophysiological and neurocognitive perspective. Furthermore, the role of self-efficacy should be considered when using measures of brain activity to better understand the neurobiology underlying the relationships between emotions and cognition.

There is a large body of evidence as to how people’s confidence in their capacity to express positive emotions is positively associated with self-esteem, optimism, prosociality, emotional stability, happiness, and contentment in situations of daily-life (Caprara and Steca, 2005, 2006; Caprara et al., 2013b; Bassi et al., 2018). Indeed, higher levels of perceived regulatory emotional self-efficacy are associated to several indicators of wellbeing at different ages (e.g., Busseri, 2018). Overall, the findings here pay testimony to the role that self-efficacy beliefs may play in enabling people to regulate their emotions and to benefit from their emotional experiences. As self-efficacy beliefs may be nurtured and strengthened through experiences, they might represent ideal vehicles to promote the changes in attitudes and behavior needed to best take advantage of ones’ emotions. In this respect, social cognitive theory indicates the steps one should take to design and implement effective interventions (Bandura, 1997). Recently, the programs to strengthen self-efficacy beliefs and their main sources -mastery experience, vicarious experience, verbal persuasion, and the regulation of physiological states– have been examined from a neurochemical perspective (Stone, 2018). It was highlighted that the practice to develop self-efficacy beliefs may increase the likelihood of risk/reward brain chemical release (i.e., dopamine, serotonin, oxytocin, and endorphin), while decreasing the likelihood of releasing the stress hormone cortisol.

Despite the strengths of our studies, we are aware that the use of convenience samples has obvious limitations when generalizing the differences observed in association with age and gender, and that further research will be needed to establish the robustness of the above findings, especially with a longitudinal design. While nurturing competence and self-confidence in the domains of affect regulation and of interpersonal relations represent a common challenge of clinicians and health professionals (Caprara et al., 2010b), there is still an important void to be breached regarding the physiological basis that underlie these relationships.

In examining the relationship between regulatory emotional self-efficacy and wellbeing, this study highlights the importance of investigating the common and the unique biological/neurological correlates of self-efficacy beliefs, mainly as a product of self-reflection, that are emotional and cognitive aspects of wellbeing. Further clarification of the brain structures and the physiological elements that underlie the constructs investigated would undoubtedly aid the design of future interventions and strategies to enhance an individual’s psychological and physical wellbeing.

## Data availability statement

The original contributions presented in this study are included in the article/Supplementary material, further inquiries can be directed to the corresponding author.

## Ethics statement

The studies involving human participants were reviewed and approved by the Institutional Review Board at the Madrid Open University (UDIMA). The patients/participants provided their written informed consent to participate in this study.

## Author contributions

MC and MG contributed substantially to the concept and design of the study, collected the data, and performed and interpreted the analysis. MM and IR-U contributed to the design, interpretation of the data, drafted, and revised the manuscript. All authors contributed to the data interpretation, critical and substantial revisions of the manuscript, and approved the submitted version.
