# Supplementary material for: Self-efficacy beliefs in managing positive emotions: Associations with positive affect, negative affect, and life satisfaction across gender and ages
Source: Front Hum Neurosci. 2022 Aug 8;16:927648. doi: 10.3389/fnhum.2022.927648 (PMC9393478; doi:10.3389/fnhum.2022.927648)
Supplement: Supplementary file 1 [file Data_Sheet_1.docx]

*Appendix 1: Final items to measure Self-Efficacy in managing positive emotions separately for each dimension*

|  |
| --- |
| How well can you: |
| *Perceived Self-efficacy in: Expressing Positive Emotions (SE/POS)* |
| 1. Rejoice over your successes? |
| 2. Express enjoyment freely at parties? |
| 3.Express joy when good things happen to you? |
| 4. Feel gratified overachieving what you set out to do? |
| *Perceived Self-efficacy in: Taking Advantage of Memories of Positive Experiences (SE/MEM)* |
| 5. Find comfort in remembering moments of joy when you find yourself in difficulty |
| 6. Take advantage from thinking of happy moments when you find yourself in stressful situation |
| 7. Remember great experiences and moments from your past when you are sad |
| *Perceived Self-efficacy in: Making Positive Use of one' s own Sense of Humor (SE/HUM)* |
| 8. Use your sense of humor to sustain your friends when they are feeling down |
| 9. Infect others with your sense of humor |
| 10. Overcome embarrassing and difficult situations with playful jokes |
| 11. Use your sense of humor when confronting difficult moments |
|  |
|  |
