# Supplementary material for: Self-efficacy beliefs in managing positive emotions: Associations with positive affect, negative affect, and life satisfaction across gender and ages
Source: Front Hum Neurosci. 2022 Aug 8;16:927648. doi: 10.3389/fnhum.2022.927648 (PMC9393478; doi:10.3389/fnhum.2022.927648)
Supplement: Supplementary file 2 [file Data_sheet_2.docx]

*Appendix 2. Unstandardized values of factor loadings and intercepts of the Confirmatory Factor Analyses of Self-Efficacy in managing Positive Emotions by Gender and Age*

|  | Gender Invariance | | | | | Age Invariance | | | | | |
| --- | --- | --- | --- | --- | --- | --- | --- | --- | --- | --- | --- |
|  | *Factor Loadings* | Intercepts | | | | *Factor Loadings* | | Intercepts | | | |
|  | *Men-Women* | *Men* | *Women* | | | *Young-Middle-Elder* | | *Young* | | *Middle* | *Elder* |
| Item 1 | 0.572 | 3.880 | | | | 0.576 | | 3.973 | | | |
| Item 2 | 0.484 | 3.895 | | | | 0.480 | | 3.971 | | | |
| Item 3 | 0.604 | 3.927 | | | | 0.611 | | 4.019 | | | |
| Item 4 | 0.608 | 3.821 | | | | 0.603 | | 3.916 | | | |
| Item 5 | 0.651 | 3.359 | | | | 0.600 | | 3.303 | | | |
| Item 6 | 0.620 | 3.465 | | | | 0.581 | | 3.402 | | | |
| Item 7 | 0.770 | 3.274 | | 3.428 | | 0.714 | | 3.286 | | | |
| Item 8 | 0.616 | 3.797 | | | | 0.576 | | 3.862 | | | |
| Item 9 | 0.643 | 3.593 | | | | 0.595 | | 3.578 | | 3.687 | 3.578 |
| Item 10 | 0.693 | 3.457 | | | | 0.637 | | 3.444 | | | |
| Item 11 | 0.752 | 3.325 | | | | 0.686 | | 3.300 | | | |
|  |  |  |  | |  | |  |  |  | | |
